# Acceptance Prediction for Answers on Online Health-care Community

**DOI:** 10.1186/s12859-019-3129-2

**Published:** 2019-11-25

**Authors:** Qianlong Liu, Kangenbei Liao, Kelvin Kam-fai Tsoi, Zhongyu Wei

**Affiliations:** 1 School of Data Science, Fudan University, Handan Road, Shanghai, China; 2 Jockey Club School of Public Health and Primary Care The Chinese University of Hong Kong, Hong Kong, China

**Keywords:** Online healthcare community, Deep learning, Natural language processing, Co-attention mechanism

## Abstract

**Background:**

With the development of e-Health, it plays a more and more important role in predicting whether a doctor’s answer can be accepted by a patient through online healthcare community. Unlike the previous work which focus mainly on the numerical feature, in our framework, we combine both numerical and textual information to predict the acceptance of answers. The textual information is composed of questions posted by the patients and answers posted by the doctors. To extract the textual features from them, we first trained a sentence encoder to encode a pair of question and answer into a co-dependent representation on a held-out dataset. After that,we can use it to predict the acceptance of answers by doctors.

**Results:**

Our experimental results on the real-world dataset demonstrate that by applying our model additional features from text can be extracted and the prediction can be more accurate. That’s to say, the model which take both textual features and numerical features as input performs significantly better than model which takes numerical features only on all the four metrics (Accuracy, AUC, F1-score and Recall).

**Conclusions:**

This work proposes a generic framework combining numerical features and textual features for acceptance prediction, where textual features are extracted from text based on deep learning methods firstly and can be used to achieve a better prediction results.

## Background

Recently, the online service system develop so fast and it covers many fields such as the legal field, the medical field and the education field. As a representative among them, the Online Healthcare Communities (OHCs) has been played an important role in bridging the gap between doctors and patients. Now it’s quite popular due to its convenience and accessibility. In OHCs, when a patient posts a question which describes his/her morbid conditions, there will be more than one doctors who post their suggestions under this question, it proves to be an effective way of communication between doctors and patients. Therefore, predicting whether a doctor’s answer will be accepted by the patient is critical in OHCs. First, some most relevant answers for patients could be retrieved from existing answers under the questions. Second, we can provide some suggestions for doctors on how to reply to patients’ questions more appropriately.

A promising line of research based on OHCs mainly lies in the relationships between different factors such as the mechanism of these relationships, e.g., the effect of using OHCs on the doctor-patient relationship and patient wellbeing [1], the benefits of social support exchanged in OHCs to patients’ mental health [2], the relationship between patients’ exercise activities and participation in OHCs [3]. What’s more, some researchers identified several factors that influence patients’ behaviors in OHCs, such as the source of information in OHCs [4] and IT enablers and health motivators [5]. In terms of the satisfaction of patients, which has an impact on the acceptance of answers, Liu et al. examined the individual and organizational reputation of doctors [6], and Yang et al. found that patients satisfaction is also affected by doctor-patient exchange frequency and the responding speed of doctors [7].

Even though these researches all presented promising results, they just ignored the text information which is considered to be the chief information carrier in OHCs. For instance, latent dirichlet allocation (LDA) [8] was applied in some existing works to extract text features from electronic health records (EHRs) and text in OHCs. Applying topic analysis on the cancer clinical notes [9]; mining some popular topics [10] and semantic analysis [11] based on the reviews in OHCs; identifying similar patients according to EHRs [12]; In addition, n-gram based methods was also used for infection detection based on EHRs [13].

With the development of computational biology, many disease-related genes are detected based on biological data and deep neural network [14–17]. In order to find out the relationships between diseases, the network based method is also introduced [18, 19]. As a result, it gives us a way to cope with biological data in big amount.

Recently, the deep neural network has been employed in a lot of natural language processing tasks, such as machine translation [20, 21], text generation [22, 23], question and answering [24, 25], dialogue systems [26–28], where we can encode a sequence of words into a vector through the recurrent neural networks (RNN) based model. The experimental results have demonstrated the effectiveness of encoding sentences with RNN-based models.

For convenience, we denote the features extracted from answers and questions as *textual features*, while the others (e.g., patient’s age and gender, the doctor’s answering order and title, the length of answer etc.) are denoted as *numerical features*. In our work, we combine these two types of features together. The process is listed as follows, first we trained a sentence encoder to encode a pair of question and answer into a vector, which is what we called textual features, after then we feed both textual features and numerical features into a classifier to predict whether an answer will be accepted by the patient. It’s a worthy to note that the fascinating attention mechanism (e.g., self-attention [29], co-attention [30]) could be integrated into our sentence encoder and any classifiers could be used in our framework. Experimental results demonstrated the effectiveness when applying textual features in predicting whether an answer will be accepted or not.

## Methods

### Dataset Description

The dataset are collected from a popular Online Healthcare Community (http://club.xywy.com). This community was found in 1999 and it’s one of the earliest online service system which explored and practised Internet healthcare services. In addition, the number of registered users exceeded 120 million and the number of daily online visitors exceed 22 million up to now. After years of development, it has been one the top platforms of online medical service industry in China.

**Fig. 1:**
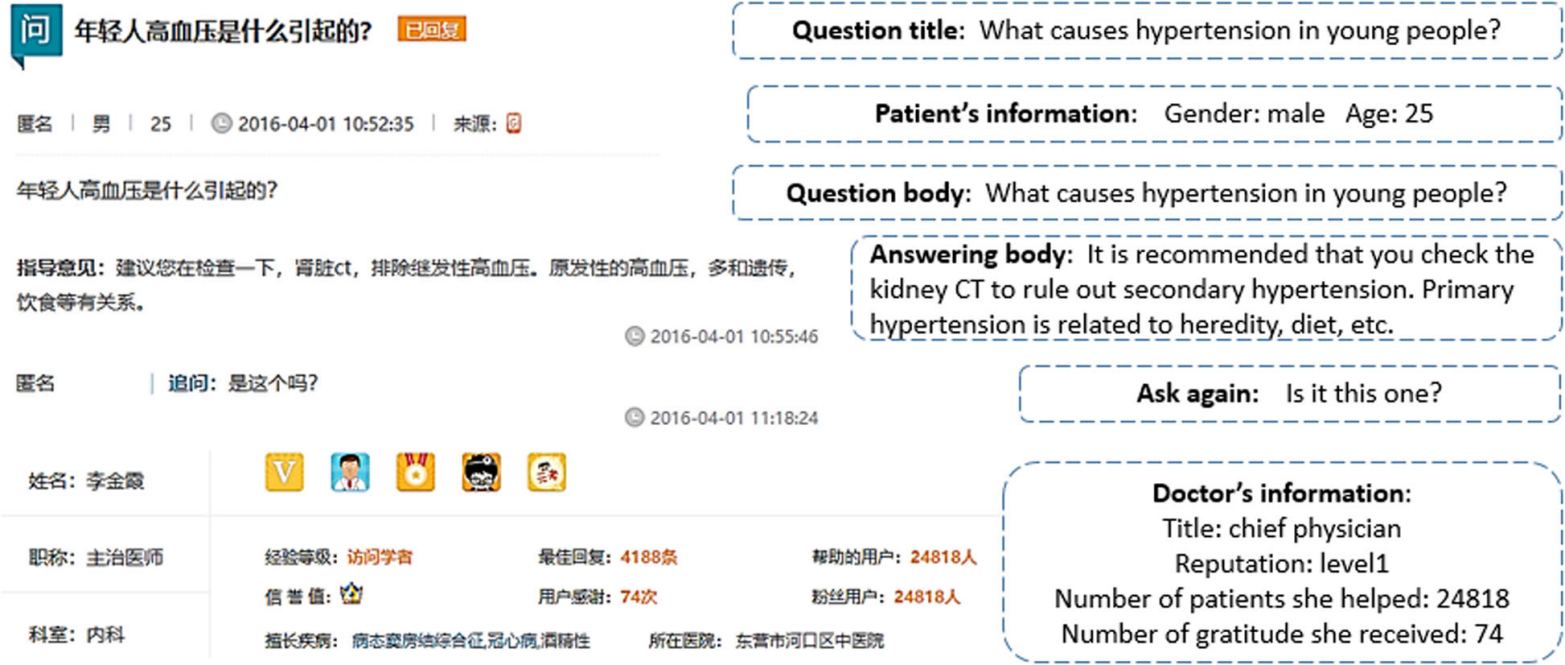
This is one example in *youwenbida* (Q&A) section, which includes patient’s information, doctor’s information, question and answering information, etc. and the details can be searched in http://club.xywy.com/ static/20160401/104740588.htm

In this website, there are many sections and most people will turn to *youwenbida* (Q&A) section for help because patients and doctors can communicate freely in this platform. There is an example in the *youwenbida* (Q&A) section and the detailed information we can get is shown in Fig. 1.

**Fig. 2:**
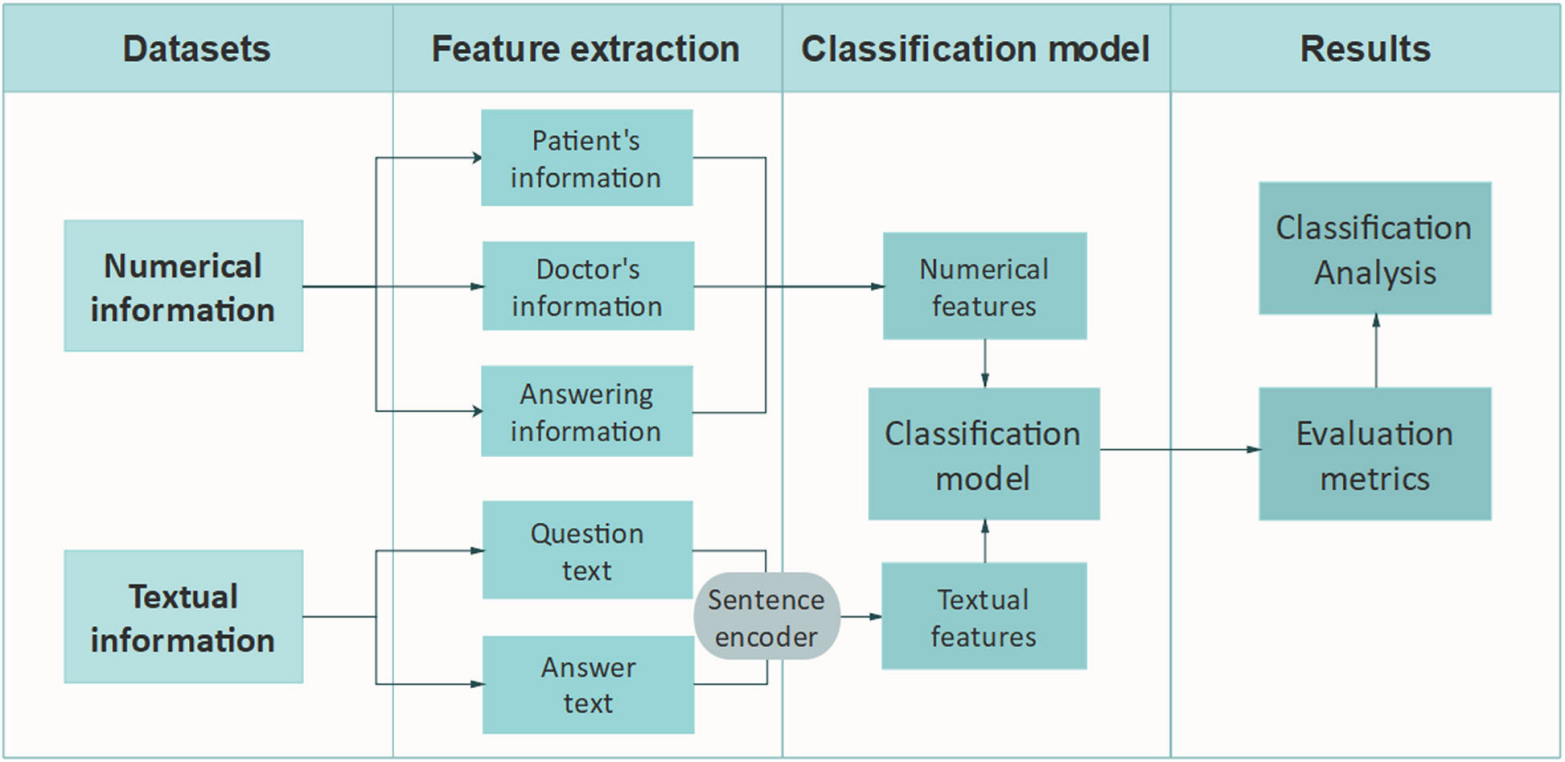
The pipeline for data preprocessing and feature extraction. textual and numerical information are extracted differently, after that the numerical features and textual features are joined together to predict the acceptance of answers. The details of sentence encoder are shown in Fig. 3

On this platform, the process is listed as follows. A patient could post a question which depict his/her morbid condition in words, then there will be more than one doctor who give some suggestions to the patient under this question. After that, the patient can either accept one of the suggestions or inquire some specific doctors again until his/her question is solved. We denote one suggestion is helpful to the patient only if he/she adopts this suggestion or inquire after this suggestion again. The pipeline for data processing and model construction can be seen in Fig. 2.

**Fig. 3:**
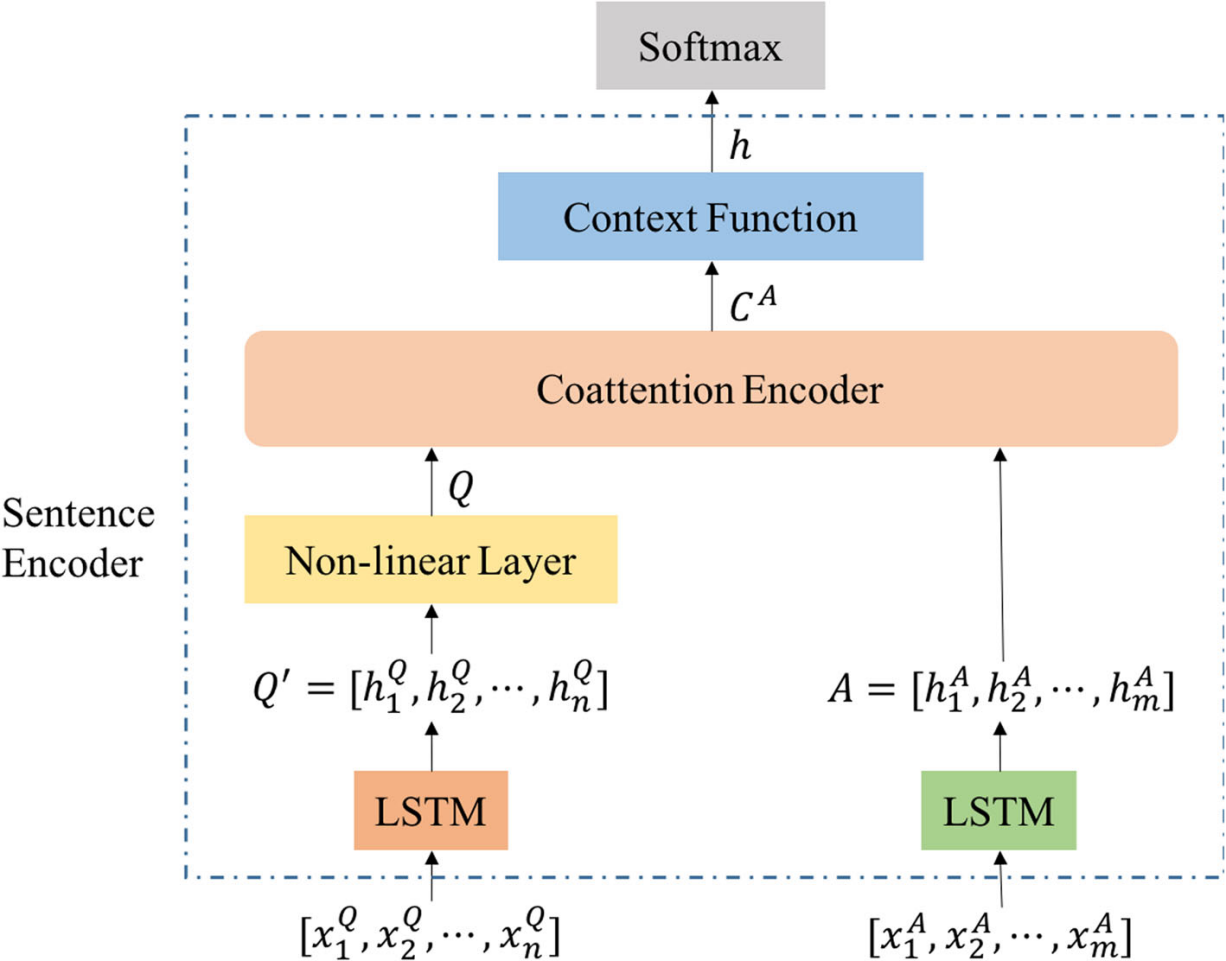
Architecture of our sentence encoder. The question and answer are first encoded by LSTM and no-linear layer as *Q* and *A* respectively, then *Q* and *A* are encoded by co-attention encoder [30] as *C*^*A*^. Next, context function maps *C*^*A*^ into a vector *h*, i.e, the representation of the given pair of question and answer, which are fed into softmax layer for binary classification, i.e., accepted or not

The dataset we crawled from the website contains both numerical and textual features. The numerical features contains three parts: patient’s information, doctor’s information and answering information. These features just play an important role for us to predict whether an answer will be helpful to the patient.

We list all the numerical features that will be used in our model in Table 1. Among them, some are categorical variables so we change them into one-hot vector, while for the continuous variable, we take logarithmic transformation because they all follow the right-skewd distribution.

**Table 1:** Numerical features used in the model

	Feature	Remarks
Patient’s information	Patient’s age	integer, 0-98
	Patient’s gender	categorical: male/female
Doctor’s information	Doctor’s title	categorical: chief physician/
		attending physician/assistant physician
	Doctor’s reputation	categorical: level1/level2/level3
	Number of patients the doctor helped	integer, 0-406342
	Number of gratitude the doctor received	integer, 0-13883
Answering information	Number of Chinese characters in the answer	integer, 12-734
	Time difference between question and answer	continuous (in seconds), 0-8005.37
	Answer’s order under the corresponding question	integer, 1-25

**Patient’s information:** Each patient is required to fill their personal information once they register on the website. And the information that we can see is their age and gender, which are included in our numerical features.

**Doctor’s information:** Doctors in OHCs are strictly certified so they have specific information on the website. The doctor’s information we used includes doctor’s title, doctor’s reputation, the number of online patients this doctor has helped, the number of gratitude received from online patients.

**Answering information:** The answering information comes from interactions between patients and doctors, which reflect the corresponding behaviors of doctors and influence patient’s satisfaction in turn [7]. The answering information includes the length of answer and doctor’s answering order under the corresponding question.

**Textual information:** The textual information are mainly extracted from patient’s questions and the doctor’s answers, while we can only cope with it through natural language processing. And the details of extracting textual features will be described in next section.

#### Data Preprocessing

##### Numerical data preprocessing

In our dataset, we collected totally 225 kinds of diseases and nearly 17 million records. For the ease of calculation, we select two kinds of diseases as representatives, one is hypertension which is a representative of chronic disease, another one is Oral Ulcer which is a representative of acute disease.

Due to the fact that only when a patient respond to answer can we know whether an answer is helpful to the patient, so we just exclude the records where the patient has not any response and keep the others. Among all the answers under a question, if the patient think it’s helpful then we label it as the positive record, while for the other answers we label them as the negative records. Moreover, we exclude the records with missing values.

In the processed dataset, there are 15014 samples in total. Among them, nearly 60% samples are positive and nearly 40% samples are negative. Moreover, the samples of hypertension account for about 90% of total samples and the samples of Oral ulcer account for about 10% of total samples. There is a more detailed description of this dataset in Table 2.

**Table 2:** Description of textual information

Marker	Number
average # of Chinese characters in questions	73.6
average # of Chinese characters in answers	94.0
average # of answers per question	2.36
# of patients	6352
# of doctors	1394

##### Textual data preprocessing

Because All the text we obtained in our dataset is in Chinese, at first we use Jieba (https://github.com/fxsjy/jieba) in Python to perform text segmentation in that Jieba is quite a popular module for Chinese word segmentation. Moreover, in order to distinguish the uncommon words (e.g., disease names, drug names) better, some customized dictionaries are added in the original dictionary, such as the medical thesaurus from *Sogou Input* (https://pinyin.sogou.com/dict/) and *QQ Input* (http://cdict.qq.pinyin.cn/). After then, there are totally 385950 words are added to the original dictionary.

After text segmentation, we remove the stop words from the text, which contain modal words, conjunctions and pronouns etc. Then, we perform word embedding so that we can use it in the recurrent neural network (The preprocessing is performed on whole dataset, i.e., 17 million records).

### Textual Features Extraction

**Sentence Encoder** In order to get a representation from the text information, we apply the sentence encoder to encode both the questions and answers. Because of the strong relationship between an answer and its corresponding question, we need a model to join them together and output a joint representation of them. Hence we apply the co-attention mechanism [30] and get the joint representation. The framework of sentence encoder is described in Fig. 3.

Here is a brief introduction to this process. When given a pair of question and answer, we denote $\left (x_{i,1}^{Q}, x_{i,2}^{Q},..., x_{i,n}^{Q}\right)$ as the sequence of question words and $\left (x_{i,1}^{A}, x_{i,2}^{A},...,x_{i,m}^{A}\right)$ as the sequence of answer words. Through the LSTM layer, the question words can be encoded as $Q'=\left [h_{1}^{Q},h_{2}^{Q},\ldots,h_{n}^{Q}\right ]\in \mathbb {R}^{l\times n}$ and the answer words can be encoded as $A=\left [h_{1}^{A},h_{2}^{A},\ldots,h_{m}^{A}\right ]\in \mathbb {R}^{l\times m}$, where $h_{t}^{Q}=\text {LSTM}_{Q}\left (h_{t-1}^{Q}, x_{t}^{Q}\right)$, $h_{t}^{A}=\text {LSTM}_{A}\left (h_{t-1}^{A},x_{t}^{A}\right)$ and *l* is the state size of LSTM. LSTM_*Q*_ and LSTM_*A*_ could share parameters for the same representation power. In addition, we introduce a non-linear projection layer to map the question into a different space of answer A. Finally the question can be represented as $Q=\text {tanh}(W^{Q}Q^{'}+b^{Q})\phantom {\dot {i}\!}$.

In order to calculate the affinity scores which corresponds to all pairs of document words and quetion words, we compute the affinity matrix as follows: $L=A^{\top }Q\in \mathbb {R}^{m\times n}$. Then we apply the row-wise normalization in L to get the attention weights across the answers for each word in the question and the score matrix *S*^*Q*^ is generated. Similarly, when applying the column-wise normalization in L, the score matrix *S*^*A*^ can be generated. The calculation formula are listed as follows:

1 $$\begin{array}{@{}rcl@{}}  S^{Q}&=&\text{sotfmax}(L)\in \mathbb{R}^{m\times n} \\ S^{A}&=&\text{sotfmax}(L^{\top})\in \mathbb{R}^{n\times m}  \end{array} $$

After that, we compute the co-dependent representation of the question and answer as Xiong et al. [30] did:

2 $$ \hspace{48pt} C^{A}=[Q;C^{Q}]S^{A}\in \mathbb{R}^{2l\ \times m}  $$

where the notation [a; b] is the horizontal concatenation of the vectors a and b. $C^{Q}=AS^{Q}\in \mathbb {R}^{l\times n}$ is the summaries of the question that attends each word in the answer. Then a pair of question and answer can be encoded as *C*^*A*^. In order to extract more useful information in acceptance prediction, we train a sentence encoder. More specifically, when given a positive pair of question and answer (*Q*_*i*_,*A*_*i*_), we aim to maximize the accepted probability of acceptance if the answer for this question was accepted. Otherwise, the objective is to minimize the accepted probability. Then we apply the cross-entropy as the loss function and write it as follow:

3 $$ \begin{aligned} \mathcal{L} =&-\sum_{i}^{N}[y_{i}\log p(\hat{y}_{i}=1|Q_{i},A_{i})\\ &+(1-y_{i})\log p(\hat{y}_{i}=0|Q_{i},A_{i})] \end{aligned}   $$

where *N* is the total number of pairs of questions and answers.

In order to get the acceptance probability of an answer, we use a context function which maps *C*^*A*^ into a vector and the softmax layer could be applied to compute the acceptance probability. Actually we can regard each column *c*_*i*_ in *C*^*A*^ as the encoding vector for the *i*-th word of answer. In addition, we can use another LSTM layer *f*_*C*_ here and outputs the last hidden state as the representation of the pair of question and answer. Moreover, we could also use a multi-layer perceptron (MLP) as *f*_*C*_ by applying it on each column of *C*^*A*^. Finally a vector of length m will be generated to represent h.

**Acceptance Prediction** After the construction of the sentence encoder above, we can extract the textual features when we are given a pair of question and answer (*Q*_*i*_,*A*_*i*_) and the formula is as follow: $h_{i}^{text}=sentenceEncoder(Q_{i}, A_{i})$. Because we can also get the numerical features from the doctor, the patient and their corresponding behaviors, a dataset ${} \{(h_{i}, y_{i})\}_{i=1}^{N}$ can be built, where $h_{i}=\left (h_{i}^{text}, h_{i}^{num}\right)$ and *y*_*i*_ indicates whether answer *A*_*i*_ is helpful to the patient on question *Q*_*i*_.

### Experimental Setup

As we discussed in “Methods” section, all sentences in our dataset are segmented and stop words are removed. In order to feed the sentences of question and answer into sentence encoder, we first use the word embedding method to change them into vectors based on the complete dataset of 17 million records. To realize word embedding, the word2vec [31] algorithm is applied and the dimension of vectors is set to 100. In addition, if the frequency of the words is below 30 and we replace them with ’UNK’. Among the whole dataset, we use 80% of them to train the sentence encoder and the rest 20% is used for testing, the classifier is built with a 5-fold cross validation. The objective of the sentence encoder is to minimize the loss in Eq. (3) on the held-out dataset. In addition, some parameters in our model is set as follows: the state size in the LSTM cell is set to 100, the batch size is set to 100 and the learning rate is set to 0.001. Moreover, we also adopt the dropout module and gradient clipping module in our model to avoid over fitting.

**Model comparisons.** After the training of sentence encoder, the textual features of each sample in non-held-out set can be extracted by forward passing through the sentence encoder. In order to evaluate the sentence encoder, we trained a gradient boosting classifier (GBC).There are two types of sentence encoders that we will use for comparisons, one is LSTM and it will input the whole sentence and output the last state as the final textual features, we denote it as LSTM-GBC. Another one is MLP and it can also outputs a vector $h\in \mathbb {R}^{m}$, we denote it as MLP-GBC. First, in order to compare the models with different encoders we will compare the result between LSTM-GBC and MLP-GBC. Second, in order to test the effectiveness of textual features and numerical features in acceptance prediction, we will compare these three models: the model with textual features only, the model with numerical features only and the model with both numerical and textual features.

**Evaluation metrics.** So as to evaluate the performance of different models, we use some common metrics such as Recall, Precision, Accuracy, AUC and F1-score. The definitions of these metrics are listed in Table 3.

**Table 3:** Definition of metrics

metric	definition
Recall	*TP*/(*TP*+*FN*)
Precision	*TP*/(*TP*+*FP*)
Accuracy	(*TP*+*TN*)/(*TP*+*TN*+*FP*+*FN*)
F1-score	2×(*Reacall*×*Precision*)/(*Reacall*+*Precision*)
AUC	The area under ROC curve

In Table 3, *TP* means true positives, *TN* means true negatives, *FP* means false positives and *FN* means false negatives. ROC curve is plotted with the *FP* *rate* as the horizontal axis and the *TP* *rate* as the vertical axis. The *FP* *rate* and *TP* *rate* are calculated as follows:

$$FP~rate=\frac{TP}{TP+FN},\quad TP~rate=\frac{FP}{FP+TN} $$

## Results

**Model Results.** The experimental results are listed in Table 4. We can see that the model with numerical features only outperforms the model with textual features only. While the model with both textual and numerical features has a better performance in nearly all metrics compared with the model with numerical features only. It shows that the additional textual features can significantly improve the model’s ability in acceptance prediction and it demonstrates that the sentence encoder can extract the textual from text effectively. When comparing the sentence encoder based on LSTM with the sentence encoder based on MLP, we can see if we only consider the textual features, LSTM-GBC performs better than MLP-GBC, while when we consider both textual and numerical features, MLP-GBC achieves a better result. We attribute the unstable results to the small amount of dataset for training the sentence encoder.

**Table 4:** Model results

	Model	F1-score	AUC	Accuracy	Recall	Precision
Textual features	LSTM-GBC	0.396 ±0.022	0.626 ±0.020	0.628 ±0.011	0.313 ±0.022	0.540 ±0.025
	MLP-GBC	0.349 ±0.008	0.619 ±0.016	0.627 ±0.009	0.257 ±0.006	0.545 ±0.027
Numerical features	GBC	0.729 ±0.015	0.859 ±0.015	0.803 ±0.009	0.677 ±0.027	**0.791** ±0.015
All features	LSTM-GBC	0.734 ±0.023	0.862 ±0.012	0.804 ±0.016	0.694 ±0.025	0.779 ±0.023
	MLP-GBC	**0.746** ±0.011	**0.864** ±0.010	**0.809** ±0.009	**0.721** ±0.009	0.772 ±0.019

the significance means for each metric, which model performs better than the other models

**Fig. 4:**
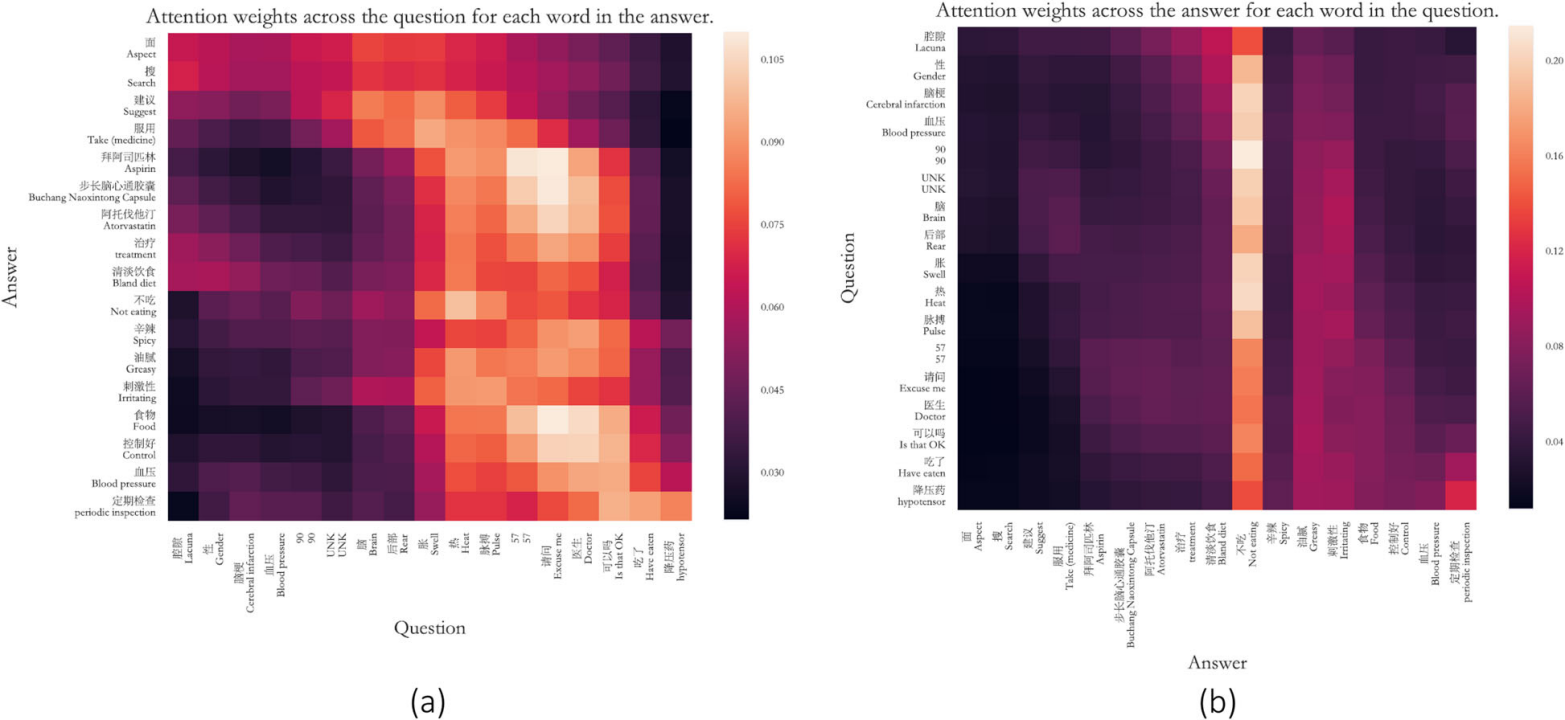
An example of attention weights of a question and the accepted answer. **a** The attention weights across the question for each word in the answer. **b** The attention weights across the answer for each word in the question. Stop words in the question and answer text are removed and out of vocabulary words are replaced with UNK. The web page of this question and answer is available at http://club.xywy.com/static/20160111/99078139.htm

**Attention Analysis.** Figure 4 shows the attention weights (i.e., score matrix *S*^*A*^ and *S*^*Q*^). From Figure 4 (a), we can see that the accepted answer is mainly focus on the uncertain part of the question, such as “Is that OK?”,“Excuse me”, which means the doctors will answer the questions with more uncertain part. Figure 4 (b) shows that the accepted answer is mainly focus on “no eating”, which indicates that the patients is prone to accept an answer with diet suggestions. In conclusion, the sentence encoder, which is enhanced with co-attention mechanism, is able to extract useful textual features attending on both question and answer for a better acceptance prediction.

## Discussion

In this work, we leverage both textual features and numerical features to predict the acceptance for answers in OHCs and demonstrate the procedure of textual features extraction, which provides a guideline to text involved prediction tasks. Moreover, any other deep learning methods for natural language processing can be integrated into this framework to extract textual features automatically for a better results. Also, there are several limitations. First, we train the sentence encoder only for two kinds of diseases in this work, and the generalization to other diseases of the sentence encoder still needs to be verified. Second, we can only extract textual features based on a manually constructed word list because of the lack in Chinese medical resources, which limits the performance of our methods.

## Conclusion

In this work, in order to predict the acceptance of answers, we propose a framework which combine both the numerical and textual features. Meanwhile, the sentence encoder is also introduced to extract the textual features from the texts. The experimental results demonstrated that additional textual features can significantly improve the model’s ability in acceptance prediction and the sentence encoder can extract the textual from text effectively. In the future, we will try to use some state-of-the-art sequence models and the reinforcement learning based models to extract textual features from text.

## Data Availability

The data cannot be made public because of security policies.

## Abbreviations

AUC: Area Under Curve
EHRs: Electronic Health Records
GBC: Gradient Boosting Classifier
LSTM: Long Short-Term Memory
MLP: Multi-Layer Perceptron
OHC: Online Healthcare Communities
RNN: Recurrent Neural Network
ROC: Receiver Operating Characteristic

## References

[1] Liu X, Liu QB, Guo X. Patients’ use of social media improves doctor-patient relationship and patient well being: evidence from a natural experiment in china. In: Thirty Seventh International Conference on Information Systems. Dublin: 2016.

[2] Yan L, Tan Y. Feeling blue so going online: an empirical study on effectiveness of virtual social networking. Workshop on Health IT and Economics (WHITE). Washington: 2010a.

[3] Ba S, Wang L (2013). Digital health communities: The effect of their motivation mechanisms. Decis Support Syst.

[4] Yang H, Guo X, Wu T, Ju X (2015). Exploring the effects of patient-generated and system-generated information on patients’ online search, evaluation and decision. Electron Commer Res Appl.

[5] Xiao N, Sharman R, Rao HR, Upadhyaya S (2014). Factors influencing online health information search: An empirical analysis of a national cancer-related survey. Decis Support Syst.

[6] Liu X, Guo X, Wu H, Wu T (2016). The impact of individual and organizational reputation on physicians’ appointments online. Int J Electron Commer.

[7] Yang H, Guo X, Wu T (2015). Exploring the influence of the online physician service delivery process on patient satisfaction. Decis Support Syst.

[8] Blei DM, Ng AY, Jordan MI (2003). Latent dirichlet allocation. J Mach Learn Res.

[9] Chan KR, Lou X, Karaletsos T, Crosbie C, Gardos S, Artz D, Ratsch G. An empirical analysis of topic modeling for mining cancer clinical notes. In: 2013 IEEE 13th International Conference on Data Mining Workshops. IEEE: 2013. p. 56–63. 10.1109/icdmw.2013.91.

[10] Hao H, Zhang K (2016). The voice of chinese health consumers: a text mining approach to web-based physician reviews. J Med Internet Res.

[11] Paul MJ, Wallace BC, Dredze M (2013). What affects patient (dis) satisfaction? analyzing online doctor ratings with a joint topic-sentiment model. AAAI Workshop on Expanding the Boundaries of Health Informatics Using AI.

[12] Arnold CW, El-Saden SM, Bui AA, Taira R (2010). Clinical case-based retrieval using latent topic analysis. AMIA Annual Symposium Proceedings.

[13] Tou H, Yao L, Wei Z, Zhuang X, Zhang B (2018). Automatic infection detection based on electronic medical records. BMC Bioinformatics.

[14] Peng J, Guan J, Shang X (2019). Predicting parkinson’s disease genes based on node2vec and autoencoder. Front Genet.

[15] Peng Jiajie, Hui Weiwei, Li Qianqian, Chen Bolin, Hao Jianye, Jiang Qinghua, Shang Xuequn, Wei Zhongyu (2019). A learning-based framework for miRNA-disease association identification using neural networks. Bioinformatics.

[16] Cheng L, Wang P, Tian R (2019). Lncrna2target v2.0: a comprehensive database for target genes of lncrnas in human and mouse. Nucleic Acids Res.

[17] Yang H, Zhao T, Zang T, Zhang Y, Cheng L (2018). Identification of alzheimer’s disease-related genes based on data integration method. Front Genet.

[18] Peng J, Zhu L, Wang Y, Chen J. Mining relationships among multiple entities in biological networks. IEEE/ACM Trans Comput Biology Bioinformatics. 2019:1–1. 10.1109/tcbb.2019.2904965. 10.1109/TCBB.2019.2904965 30872239

[19] Cheng L, Hu Y, Sun J (2018). Dincrna: a comprehensive web-based bioinformatics toolkit for exploring disease associations and ncrna function. Bioinformatics.

[20] Cho K, Gulcehre BvMC, Bahdanau D, Schwenk FBH, Bengio Y. Learning phrase representations using rnn encoder–decoder for statistical machine translation. In: Proceedings of the 2014 Conference on Empirical Methods in Natural Language Processing (EMNLP). 10.3115/v1/d14-1179.

[21] Chen MX, Firat O, Bapna A, Johnson M, Macherey W, Foster G, Jones L, Parmar N, Schuster M, Chen Z, et al.The best of both worlds: Combining recent advances in neural machine translation. arXiv preprint arXiv:1804.09849. 2018.

[22] Li J, Luong M-T, Jurafsky D. A hierarchical neural autoencoder for paragraphs and documents. arXiv preprint arXiv:1506.01057. 2015.

[23] Donahue J, Anne Hendricks L, Guadarrama S, Rohrbach M, Venugopalan S, Saenko K, Darrell T. Long-term recurrent convolutional networks for visual recognition and description. In: 2015 IEEE Conference on Computer Vision and Pattern Recognition (CVPR): 2015. p. 2625–34. 10.1109/cvpr.2015.7298878. 10.1109/TPAMI.2016.2599174 27608449

[24] Chen D, Fisch A, Weston J, Bordes A. Reading wikipedia to answer open-domain questions. In: Proceedings of the 55th Annual Meeting of the Association for Computational Linguistics (Volume 1: Long Papers): 2017. p. 1870–9. 10.18653/v1/p17-1171.

[25] Tay Y, Tuan LA, Hui SC. Cross temporal recurrent networks for ranking question answer pairs. arXiv preprint arXiv:1711.07656. 2017.

[26] Wei Z, Liu Q, Peng B, Tou H, Chen T, Huang X, Wong K-F, Dai X. Task-oriented dialogue system for automatic diagnosis. In: Proceedings of the 56th Annual Meeting of the Association for Computational Linguistics (Volume 2: Short Papers): 2018. p. 201–7. 10.18653/v1/p18-2033.

[27] Peng B, Li X, Gao J, Liu J, Wong K-F. Deep dyna-q: Integrating planning for task-completion dialogue policy learning. In: Proceedings of the 56th Annual Meeting of the Association for Computational Linguistics (Volume 1: Long Papers): 2018. p. 2182–92. 10.18653/v1/p18-1203.

[28] Wen T-H, Vandyke D, Mrksic N, Gasic M, Rojas-Barahona LM, Su P-H, Ultes S, Young S. A network-based end-to-end trainable task-oriented dialogue system. arXiv preprint arXiv:1604.04562. 2016.

[29] Vaswani A, Shazeer N, Parmar N, Uszkoreit J, Jones L, Gomez AN, Kaiser Ł, Polosukhin I. Attention is all you need. In: Advances in Neural Information Processing Systems: 2017. p. 5998–6008. http://arxiv.org/abs/1706.03762.

[30] Xiong C, Zhong V, Socher R. Dynamic coattention networks for question answering. arXiv preprint arXiv:1611.01604. 2016.

[31] Mikolov T, Sutskever I, Chen K, Corrado GS, Dean J. Distributed representations of words and phrases and their compositionality. In: Advances in Neural Information Processing Systems: 2013. p. 3111–9.

